# A Current Sensing Cross-Component Induction Magnetometer for Use in Time-Domain Borehole Geophysical Electromagnetic Surveys

**DOI:** 10.3390/s25061646

**Published:** 2025-03-07

**Authors:** Joseph Hamad, James Macnae

**Affiliations:** 1 School of Applied Sciences, RMIT University, Melbourne 3001, Australia; yjhamad@gmail.com; 2 CD3D Pty Limited, Glen Iris 3146, Australia

**Keywords:** geophysics, electromagnetic, borehole, cross, magnetometer, sensors, induction, coil, ferromagnetic

## Abstract

Electromagnetic sensors are best defined by their linearity, signal sensitivity, and noise level. In borehole time-domain electromagnetics (TEM) the cross-components are defined as the two components perpendicular to the borehole’s axial direction. Induction sensors measuring voltage across an open coil for the cross-components have poor sensitivity, and fluxgate magnetometers have been a common band-limited alternative for borehole TEM surveys. In this research, we use a shorted coil with current rather than voltage sensing circuitry to produce a cross-component induction magnetometer (CCIM). With flux coupling and electronic adjustments, we achieved a low-cut corner frequency of 3.5 Hz in the final design of the CCIM. For the prototype sensor, we found the simple ratio of measured inductance L to winding resistance R to be a poor predictor of the −3 dB corner frequency, and a transfer function measurement was required. The cause of the discrepancy may be that the self-inductance measured by a meter is different from the coupling inductance to an external field. The measured noise level of our CCIM sensors was 125 pT/√Hz at 1 Hz, compared to a geometrically longer axial component sensor with 4 pT/√Hz at this frequency. However, our design matched the typical fluxgate noise level of 6 pT/√Hz at 10 Hz. Further, the CCIM sensors were superior to fluxgates at frequencies higher than 10 Hz, with an internal noise level of 0.1 pT/√Hz between 100 Hz and >20 kHz. Induction coils or magnetometers measuring the cross-component are attractive because they have excellent high-frequency bandwidth and can be included in the same downhole package with fluxgate sensors.

## 1. Introduction

Induction coil sensors with air cores or ferromagnetic cores are commonly used to measure secondary varying magnetic fields, or their time derivatives, from prospective subsurface conductors in time-domain electromagnetics (TEM) [1,2,3]. Recently, there has been considerable research into quantum sensors for magnetic fields, with promising results from, e.g., diamond NV centers and cryogenic SQUIDS, but these technologies either require further development or are very expensive in the case of SQUIDS [4,5]. The primary parameter of an induction coil sensor is its sensitivity, which affects the signal-to-noise ratio and thereby the depth of investigation [6]. Induction coil sensor sensitivity is directly proportional to the number of winding turns and the area perpendicular to the flux of the main axis, and it can be increased greatly using a highly permeable magnetic core [7]. However, sensitivity as a function of frequency is significantly limited for practical induction coil sensors for geophysical field use, whose application is subject to dimensional and weight constraints [8]. After specifying coil dimension and weight constraints, researchers have used mathematical optimization to maximize sensor sensitivity [9,10,11]. Additionally, sensor improvements and increased magnetic field sensitivity can be achieved by geometrical modifications to the ferromagnetic core [12,13].

Magnetic sensors used for TEM surveys require high sensitivity and wide bandwidth to pick up small late-time signals after transient excitation [14]. Instead of coils measuring voltage proportional to dB/dt, B field magnetic sensors are better suited to TEM surveys in mineral exploration for highly conductive copper and nickel sulfides [15] and borehole magnetometric resistivity surveys [16], even if they are band-limited as with fluxgate sensors. Borehole TEM sensors have much stricter geometrical constraints in that any sensor must be of small enough diameter to be enclosed in a pressure- and waterproof housing that in turn fits comfortably inside the borehole and preferably inside borehole casing or drill-rods. In mineral exploration, drill hole diameters are set by geological and mechanical stability constraints [17], generally without the consideration of any geophysical sensor diameter that might subsequently be used to log the hole. The most sensitive B field sensors, specifically SQUIDS, are not well suited for borehole deployment due to volume and pressure constraints affecting the cryostats needed for superconductive operation [14].

A borehole induction magnetometer sensor requires construction in the shape of a long thin rounded rectangular or ellipsoidal coil to fit into boreholes with some margin constraints. Generally, encapsulated sensors need to be less than 35 mm in diameter to fit through the core barrel of a BQ-sized drilling rig [17]. While BQ drill holes commonly used in mineral exploration have an outside diameter of 60 mm and an inner diameter of 46 mm, the core diameter and inner diameter of the core barrel are 36.5 mm. The sensors’ component parallel to the borehole can utilize a long ferromagnetic core to increase sensitivity, thereby resolving the sensitivity issues for geometrical constraints in the borehole. The two components perpendicular to the borehole are known as the cross-components because the cross product of these components is just the component along the borehole [18].

The Crone Geophysics cross-component EM coil sensor utilizes a short ferrite core [19]. Other sensors such as SlimBoris [20], have mostly trade-secret designs, but are expected to be air-cored because of negligible sensitivity enhancement due to de-magnetization effects in any short ferrite or ferromagnetic core [21]. Because of the low cross-component sensitivity of coils, fluxgate sensors are more commonly used for cross-component detection in TEM borehole surveys where good conductors such as nickel sulfides are the target. Several commercially available fluxgate sensors achieve a noise level of 6 pT/√Hz at 1 Hz and are linear to about 4 kHz but lack any high-frequency information due to their low-pass transfer function [22]. Recently, authors have combined the fluxgate and induction coil into one package [23,24].

A proposed but, to our knowledge, untested method to enhance sensitivity for a cross-component ferromagnetic structure was suggested by [12]. The ferromagnetic helix was predicted in modeling in [12] to permit a reduction in coil size and number of turns, thereby reducing sensor internal noise in the same manner as a ferromagnetic core reduces noise in an axial sensor.

To maximize sensor sensitivity within borehole diameter constraints, we modified the magnetic flux concentrating geometry of “dumbbell”-shaped magnetic cores, which are used in magnetic energy harvesting [25]. Essentially, we modified the end extensions to fit the cylindrical size constraints of mineral exploration boreholes. We elected to operate the sensor in closed-loop current sensing mode [26]; this is achieved using a current-to-voltage converter [27,28,29]. Operating a compact sensor in current mode requires fewer turns of wire for comparable sensitivity, thereby achieving sensor compactness and self-capacitance reductions. A limitation of current mode sensors and available operational amplifiers is large DC offsets on the output if too few turns or too thick wire are used resulting in a low coil impedance [26]. A notable advantage when an induction coil is connected to a current-to-voltage converter with an operational amplifier, the effect of stray capacitance of the coil is largely eliminated and the frequency range of the induction coil is significantly expanded [7].

In this article, we describe electromagnetic sensors to measure the cross-magnetic components in borehole time-domain electromagnetic geophysical surveys, whose components are commonly named U and V in geophysical terminology [18]. The article is organized as follows. Section 2 presents induction magnetometer concepts and the design aim. Section 3 illustrates our proposed novel design and details how the geometry enhances the flux. Section 4 presents our results, and Section 5 concludes this article.

## 2. Inductive and Other Magnetic EM Sensors

### 2.1. Sensor Considerations

There are several pros and cons of different “standard” magnetic component sensors for measuring electromagnetic signals in boreholes 1 Hz to 50 kHz in bandwidth that we considered before undertaking this project. The primary factors under consideration were noise level, bandwidth, and size. These are summarized in Table 1 from the perspective of borehole sensor design.

**Figure 1:**
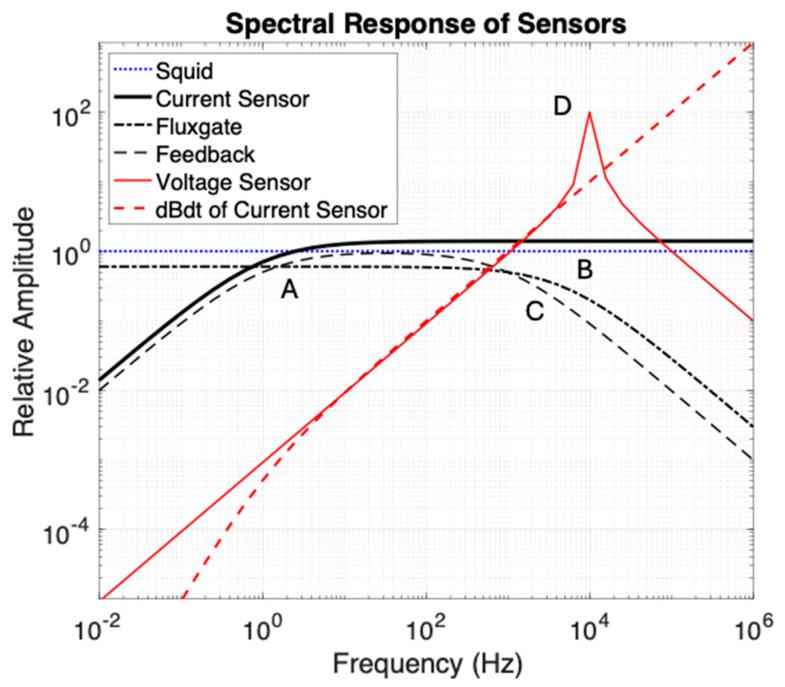
Schematic frequency response of common geophysical electromagnetic B and dB/dt field sensors. Amplitudes have been shifted vertically to separate plotted curves, as any sensor can have its gain adjusted through amplification. Symbols A to D are described in the text.

Figure 1 presents schematically the relative amplitude vs. frequency (sometimes called the spectral response) shape of several EM sensors in common geophysical usage, with the caveat that corner frequencies do vary as a function of sensor size and electronic circuit design. SQUID sensors are very small, have the best signal/noise ratio, and have an ideal flat frequency response over the region of interest. However, the cryostats needed to maintain superconducting temperatures are “too big to fit”, and the issue of containing cryogenic fluid boiloff under the high pressures at depth in a borehole eliminated this methodology from our consideration. Induction coils were the first successful borehole EM developments with very useful axial component sensors but much noisier cross-components [34]. Open-loop induction coils detecting voltage have a resonance (at symbol D in Figure 1) and very low responses at low frequencies (low sensitivity to long time constants). Fluxgates have a good low-frequency response, and despite poor signal/noise levels [35,36] and a high frequency cutoff in the low-kHz band (shown at symbol B), they are the main geophysical tools used in exploration for deep nickel targets [31]. Feedback coils have a limited bandwidth, with response drop-off at both low (A) and high (C) frequencies. These coils typically have a flat response over about 3 decades in frequency, with longer and heavier coils needed to achieve lower frequencies. Current sensing induction coils have a flat response above a low corner (A) and commonly have slightly lower noise than open-loop and feedback induction coils [21]. The main challenge in current sensor design is to achieve a low-enough corner frequency to measure slow decays, specifically in space-constrained CCIM coils. This is the architecture that we chose to research, and which is the subject of this paper. The current sensing coil output voltage can be differentiated in a parallel analog circuit and outperforms a coil voltage sensor at high frequencies (above D) with a faster falloff in sensitivity below a low corner frequency A.

### 2.2. Sensitivity of a Wideband Magnetometer Using Current-to-Voltage Conversion

**Figure 2:**
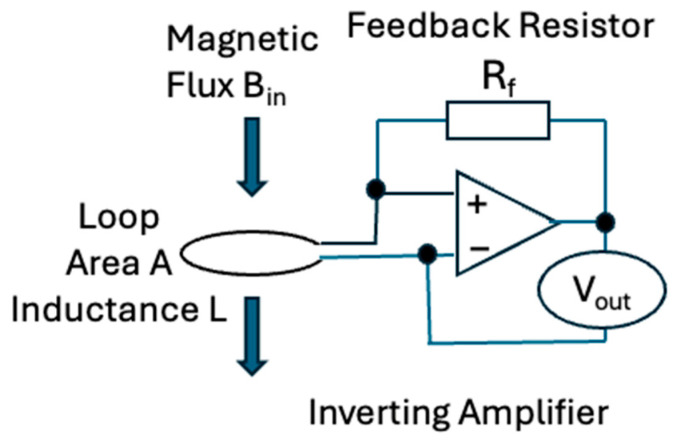
Key parameters needed to define the sensitivity of a current-to-voltage converter operated as a B field sensor using a single-turn loop. Lenz’s law states that a current is induced in the loop to exactly oppose any changes in the magnetic field passing through it. A ferromagnetic core through the coil would increase L and collect more magnetic flux, thus increasing B_in_ compared to ambient B field.

With the aim of detecting conductors with a large time constant and having a flat spectral response, we elected to operate the sensor in current sensing mode. Current sensors of the magnetic field are “null” sensors, in that the feedback circuit drives sufficient current into the coil to exactly oppose changes in the ambient B field if these changes are of a duration significantly shorter than the time constant *τ.* Induction coil sensors are based on Faraday’s law of induction [7,21,37]. The sensitivity $S_{o}$ is defined [21] as the ratio of the voltage at the output $V_{out}$ in mV to the external magnetic field $B_{in}$ in nT, passing through the sensor, with a simplified circuit shown in Figure 2:

(1) $$S_{o}=\frac{V_{out}}{B_{in}}=\frac{R_{f}}{L}N_{T}A\;\left[\frac{mV}{nT}\right],$$

where $R_{f}$ is the feedback resistor defining the induced sensor current to measured voltage ratio; L is the self-induction of the coil; $N_{T}A$ is the effective coil area being the product of the number of turns and the average area within each turn comprising the receiver coil. Equation (1) describes the frequency-independent sensitivity above the first corner frequency discussed in the next section.

### 2.3. Sensitivity Corner Frequency

If a coil sensor or the loop shown in Figure 2 is shorted by an inverting amplifier, any impulsive or step-off current induced in it will decay exponentially with a time constant τ. The primary objective for an inductive magnetometer to operate at low frequencies is to maximize the induction coil τ, given by

(2) τ=L/R

where R is the resistance of the coil windings. Maximizing τ will equivalently minimize the coils’ low-cut corner frequency $f_{c}$. The time constant increases linearly when more turns of wire of negligible thickness are added to a coil with a fixed receiving area. This can be quantified by the self-inductance L, which for “coincident” turns increases as a function of $N_{T}^{2}$ while the resistance increases linearly by $N_{T}$. Hence, taking the ratio of these two function results in a linear increase for τ with an increase in $N_{T}$. In practice, this linear increase is only achieved to the first order, as the wire has a finite thickness and additional turns need to have larger diameters or be displaced along the coil axis; hence, both self-inductance and effective enclosed areas are no longer exactly proportional to the number of turns. An improved estimate of self-inductance L for ferrite rod cores is published in [38].

## 3. Proposed Design

### 3.1. Ferromagnetic Cores

In the axial component of a dB/dt borehole coil sensor, it is common that a ferrite core is used to funnel magnetic flux through the coil and hence increase the output voltage [21]. A rule of thumb for compact windings around the middle third of a rod core is that the total effective area *A_eff_* of the coil is increased over the factor $N_{T}$*A* by the effective relative permeability of the core [21]:

(3) $$A_{eff.}=\mu_{r}N_{T}A$$

**Figure 3:**
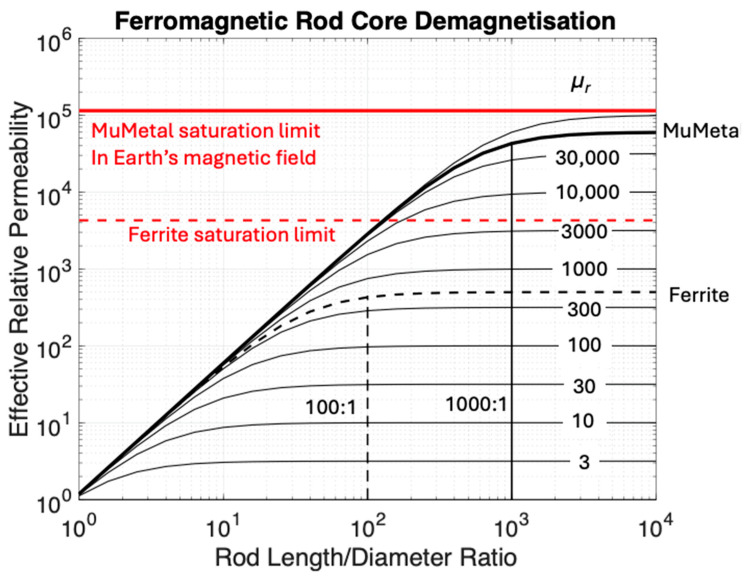
Effect of aspect ratio (length/diameter)-dependent demagnetization on effective permeability of rod cores. A ferrite core with μ_r_ = 500 achieves close to its maximum possible flux enhancement with a 100:1 aspect ratio and the collected flux is not limited by core saturation in the Earth’s magnetic field.

Effective relative permeability *μ_r_* is a function of intrinsic core permeability and geometry, specifically the aspect ratio length/diameter for rod-shaped cores [39] and to a lesser extent the coil length as a fraction of the rod. The maximum useful length of a ferrite core (Figure 3) occurs around a 100:1 aspect ratio, while effective relative permeability μ_e_ is about 90% of the intrinsic relative permeability μ_r_. The enhanced internal field in the maximum Earth’s Field Bz (say 70,000 nT) is less than μ_r_Bz = 0.035 T. With an internal saturation limit of 0.3 T, the Ferrite core should enhance the flux linearly.

Other ferromagnetic materials such as MuMetal have been used in the past for cores. Mumetal has a higher relative permeability between 60,000 and 100,000 compared to the 300 to 500 typical for ferrites and a saturation field of 0.8 T. With an aspect ratio of 1000 to 1, the flux enhancement of MuMetal would approach a factor of 50,000 for long cores (Figure 3). In practice, this enhancement is not effective at frequencies of geophysical interest due to skin-depth effects [26], where an alternating magnetic field does not penetrate the ferromagnetic material and is “shielded” from the inner parts of the core. MuMetal has a skin depth of about 0.5 mm at a frequency of 10 Hz, 50 microns at 1 kHz, and 10 microns at 25 kHz. At a 1000:1 aspect ratio (Figure 3), the maximum useful length of the core would be 10 mm, which would have a minimal flux-gathering capability. Recent developments have used laminated MuMetal with resistive layer laminations [40] to reduce the effective conductivity by minimizing the size of eddy currents, thus increasing the skin depth and hence effective length to an extent.

The ideal core for a 1 m long axial component sensor would have very high *μ*_r_ (>100,000), a high saturation flux (>1T) to be linear in the Earth’s field, and low enough conductivity (<0.5 S/m) to have a skin depth > 1 cm at 25 kHz, as well as having enough mechanical strength to support a 1000:1 aspect ratio. Since no materials could be located to meet all these specifications, a compromise is necessary. Discussion of the optimization of an axial component sensor is, however, beyond the scope of this paper.

### 3.2. Cross-Component Considerations

**Figure 4:**
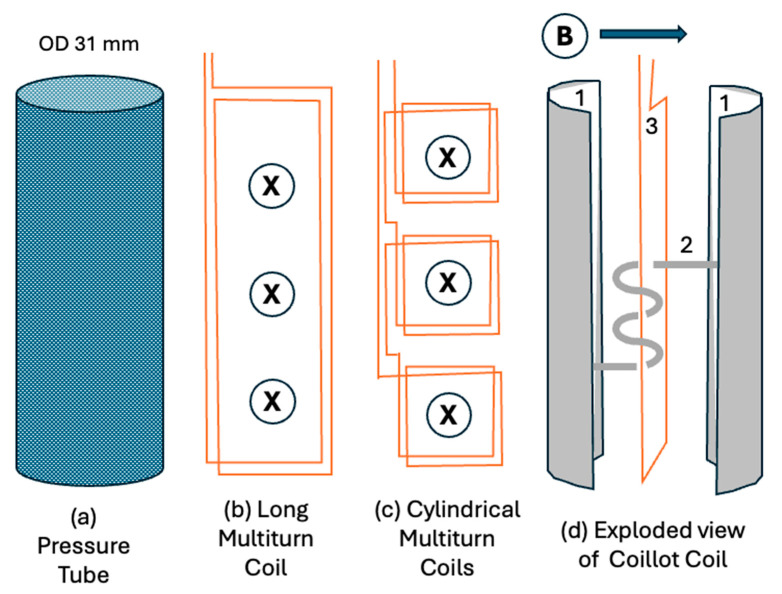
Geometry of (**a**) a pressure tube to fit through the rods in a BQ borehole. (**b**) Two turns of a multiturn coil wound to fit in the pressure tube and sense the component of magnetic field B into the page. (**c**) Multiple small multiturn coils to measure the same component. (**d**) An exploded view of the Coillot coil with a helix-wound magnetic flux channel. (1) Horizontal component ferromagnetic flux collectors or “wings” measuring B in the direction shown and (2) a flux channel consisting of a ferromagnetic multiturn tape coil passing through (3) a multiturn copper wire coil (of which only one turn is shown).

Induction magnetometers cannot have a long ferromagnetic core in the direction of measurement due to borehole width constraints. Figure 4 shows three possible sensor layouts to fit within a pressure tube housing (a) suitable for BQ boreholes. Maximizing the volume of copper wire in the coil maximizes the time constant of the shorted windings, so we determined to test one extended coil (b) as well as test stacking many smaller coils (c). The multi-coil design (c) consisted of several short, ferrite-cored cylindrical coils (20 mm diameter, 25 mm length) connected in series. These proved to have a significant number of shortcomings in that the achieved corner frequency of this design was too high to meet the required specifications. We also constructed a ferromagnetic helix-wound sensor coil, whose helical core consisted of ferromagnetic tape to repeatedly funnel magnetic flux through the copper wire coil as suggested in [12].

The wire diameter used was 0.5 mm to construct the sensors. The choice of wire diameter is not critical in terms of signal/noise optimization. Given the same overall coil dimensions, the low-cut corner frequency is a function mostly of the volume of copper forming the windings. The reason for this is that in a fixed volume, the maximum number of turns *N_T_* is inversely proportional to *d*^2^ with *d* the wire diameter. Coil inductance is roughly proportional to *N_T_*^2^, and total resistance to *N_T_ d*^2^ = *N_T_*^2^, meaning that the *L/R* ratio and time constant are insensitive to variations in *d* to the first order for the same volume of windings. The coil resistance itself is not critical in the overall noise estimation [21].

The benefit of geometry (b) over multiple coils (c) is that a greater volume of copper wire can be wound in the limited pressure tube space. As predicted, this significantly increased the time constant of the coil, which improves sensitivity to slow electromagnetic decays. From here on, we will refer to this design in this article as a U-V component sensor or U-V CCIM, with U and V referring to two orthogonal cross-component directions. Figure 4b,c show coils measuring the U component into the page, and (c) shows a coil measuring the V component parallel to the page.

Coillot et al. [12] described a hypothetical coil sensor where the ambient magnetic field was collected and passed many times through a conventional induction coil. We will call this a Flux Multiplied Induction Coil (FMIC). However, Coillot et al. did not test this concept due to a perceived lack of flexible magnetic rods or tapes. To construct an FMIC (Figure 4d), we chose a flexible magnetic material with high initial permeability, large magnetic field saturation, low hysteresis, and which was readily available. Metglas 2714A foil tape (Metglas Inc., Conway, SC, USA) is an example that satisfies these conditions [41]; however, it exhibits a high electrical conductivity. In comparison, commonly used ferrite cores are brittle, are saturated at much lower magnetic fields than Metglas, and achieve no benefit from large aspect ratios (Figure 3). Metglas tape has a thickness of 0.004 mm, so a stack of 50 ribbons would have a thickness of 0.2 mm. With the saturation constraints, the 40 cm length of tape used to make 6 magnetic helix windings had a linear aspect ratio of 2000 to 1, but its helical geometry suggested that its effective aspect ratio was less than 150:1 and so it would not saturate in the Earth’s magnetic field (Figure 3). Given that the horizontal component of the Earth’s field typically is less than 30,000 nT, we confidently predicted that an FMIC sensor would be able to detect magnetic signals in a borehole without saturation, providing an additional margin for error. The Metglas ribbons were loosely tied together to retain flexibility and not epoxied as is commonly done to obtain resistive separation and mechanical stability in cores.

**Figure 5:**
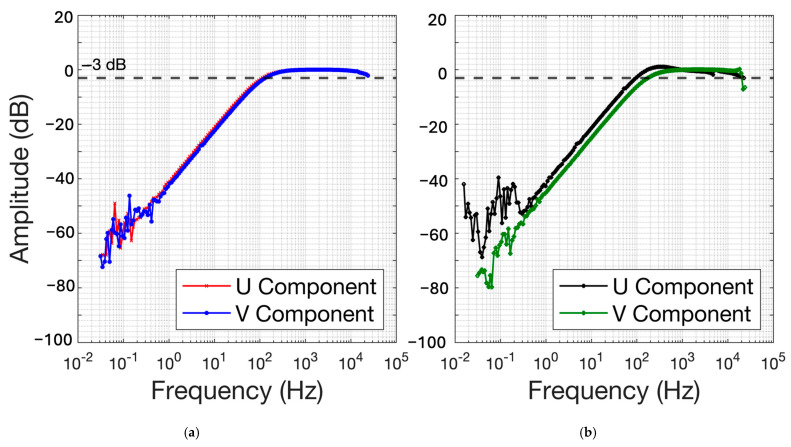
(**a**) Spectral response (amplitude vs. frequency) for two-component U and V FMIC sensor coils as proposed by [12] (**b**) Spectral response for two-component U and V air-core coil sensors without the magnetic helix. Amplitudes are scaled to unity (0 dB) at 1 kHz.

The sensing coil was wired through and around the ferromagnetic PVC pipe to form an induction coil sensor. We next experimentally determined the coil’s corner frequency, magnetic sensitivity, and internal noise. Figure 5a shows the results for the FMIC sensor with a helix-wound flux multiplier, and Figure 5b shows the results of an air-core sensor without the flux multiplier. The FMIC shown in Figure 4d only attained −3 dB low-cut corner frequencies of 113 Hz and 131 Hz for U and V components, respectively (see Section 2.3). In contrast, the simple air-core sensor coil of (b) achieved −3 dB corners of 115 Hz and 184 Hz. This is because the FMIC funnels the flux through the coil multiple times, thereby increasing the signal and thus S/N since internal noise is essentially unaffected by the flux multiplier. The effect of this signal “multiplication” is seen as reduced relative noise on the Figure 5 dB scaled plot at frequencies below 1 Hz and above 10 kHz.

**Figure 6:**
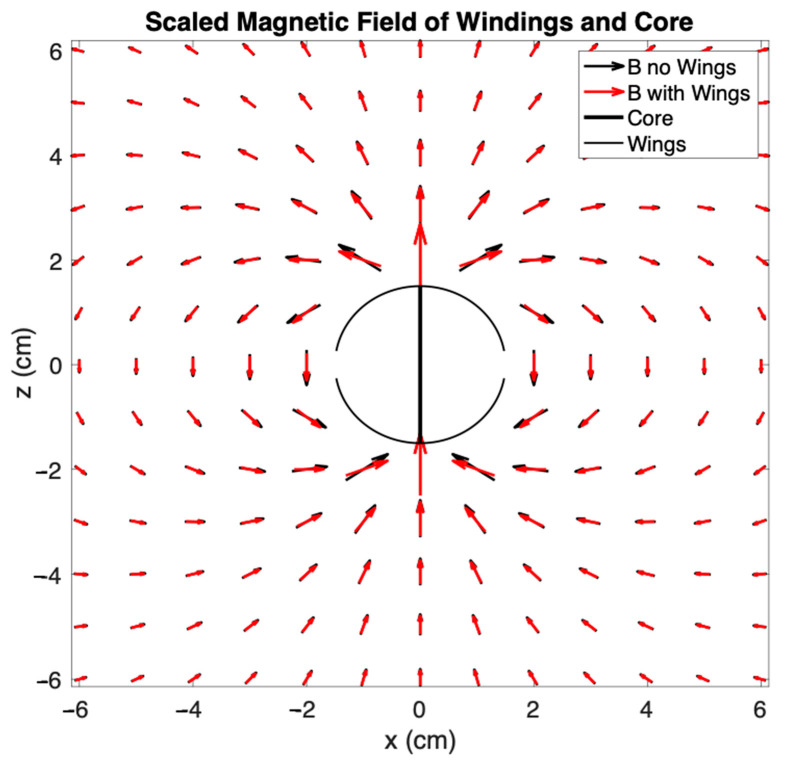
The shape of the magnetic field created by coil windings with and without flux-collection wings attached is shown schematically. Vector lengths are proportional to the cube root of amplitude.

These magnetic flux-collecting “wings” we show in Figure 4d for cross-component geometry with a single connecting core through the middle have recently been independently described in a paper discussing a magnetic field energy harvester [39]. Numerical modeling using MATLAB^®^ 2023b predicts that this core structure collects approximately 50% more flux than a thin planar flux concentrator without the wings and an FMIC should pass it through the coil multiple times. The approximation assumed that the magnetic field dipole moment distribution in the ribbon was as expected in a magnetized flat ribbon but that the direction of the local dipoles changed to follow the wings. The shape of the B field created by a ferromagnetically cored coil with and without wings is shown in Figure 6. The wings do not significantly change the shape of the flux field but have the effect of collecting more flux. We therefore included them with a central magnetic flux connection through the middle of subsequent air-cored sensors. Unlike ferromagnetic rods, the literature did not have a convenient analytical estimator for flux collection by elongated rectangular cores with wings at the top and bottom with which to compare our approximate numerical simulation. We therefore resolved to determine magnetic sensitivity and spectral characteristics experimentally.

## 4. Results

### 4.1. Sensor Construction

**Figure 7:**
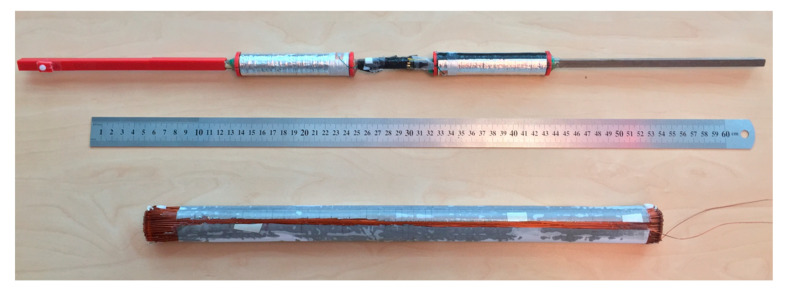
Constructed axial component sensor (**top**) and channel-wound borehole U or V CCIM (**bottom**) sensor. From the image, the direction of the received magnetic field is left or right for the axial component sensor and upwards or downwards for the imaged CCIM coil. Electrical field shielding is provided by open conductive foil wrapping.

As stated in Section 2.2, the aim was to construct a low-noise sensor that minimized the current sensor coils’ −3 dB low-cut frequency. We assembled two borehole inductive magnetometers to verify our design. The first sensor assembled was an axial component sensor using a ferromagnetic core. Since the physics of the axial component sensor is well understood in the literature [31], we used the axial component sensor for verification of our test procedures. The subsequent sensor coils we assembled are the proposed U-V CCIM coils as described in Section 3. Enameled copper wire with a diameter of 0.5 mm was used for the windings. We managed to wind 900 turns, which produced a very tight fit into the pressure tube casing. One axial and one CCIM sensor coil are shown in Figure 7.

### 4.2. Corner Frequency Measurements

To determine the predicted coils’ −3 dB low-cut frequency (Equation (2)) requires that the coils’ transfer function be measured. Our experience is that simple multimeter measurements of inductance L and resistance R do not accurately predict the corner of an “active” sensor. Transfer function estimations were achieved by measuring the spectral response of a sensor coil centered inside an air-core multiturn transmitter. To fit inside a cylindrical Gauss chamber, the axial component of the transmitter was 300 mm in diameter solenoid, whereas a 700 by 300 mm multiturn rectangular loop was used to create an energizing field for the induction magnetometers. The transmitter loop current is magnetically coupled to the sensor, with the pair electromagnetically shielded from external electromagnetic noise inside the Gauss chamber. The transmitter current took the form of a repetitive time-series combination of white and pink noise, corresponding to a randomized sum of sinusoids between 0.01 Hz and 25 kHz. A 50 kHz sampling frequency National Instruments Data Acquisition system was used to stream sample the voltage across a resistor in the transmitter current path and the inductive magnetometer voltage output.

**Figure 8:**
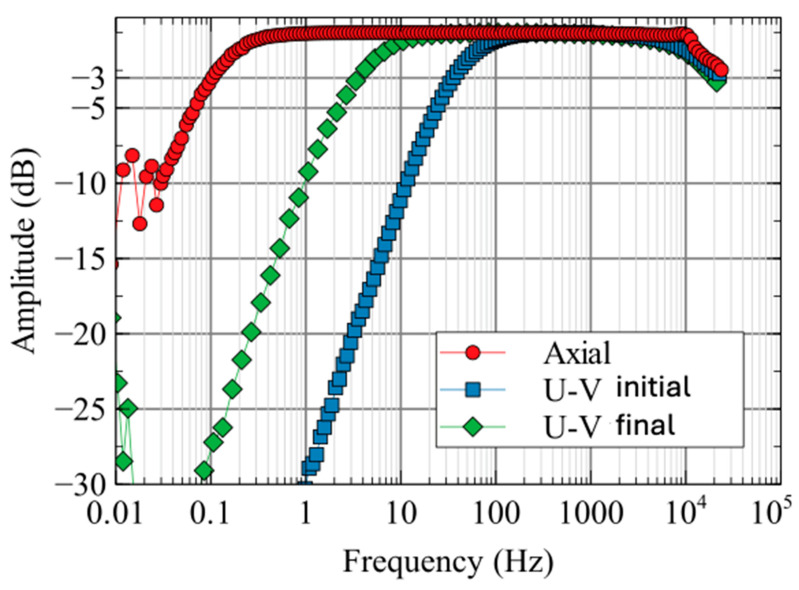
Amplitude response (ratio of output to input signal as a function of frequency) of coil sensors; the initial intrinsic U-V CCIM coil response and the final U-V CCIM coil with L/R 10:1 ratio reduction implemented. The axial component sensor response is shown with the 10:1 analog corner frequency reduction implemented. Amplitudes have been scaled to 0 dB at 1000 Hz.

Experimentally measured spectral responses for different coils are shown in Figure 8. The U-V CCIM −3 dB corner frequency with a conventional current amplifier was 35 Hz. Using proprietary components and trade-secret analog circuitry to increase the coil inductance-to-resistance ratio by a factor of 10 leads to a 10-fold reduction in the sensor corner frequency to 3.5 Hz. Frequencies below 0.1 Hz were considered unimportant for the spectral characterization and, with the chosen averaging times, had relatively large noise and a poor spectral response estimate. The response above 10 kHz shows high-cut properties just before the 25 kHz Nyquist frequency of the data acquisition system. The 75 cm long axial component sensor with an optimized current-to-voltage converter achieved a −3 dB corner frequency of 0.1 Hz and a frequency response over 5 decades between the low and high corners.

**Figure 9:**
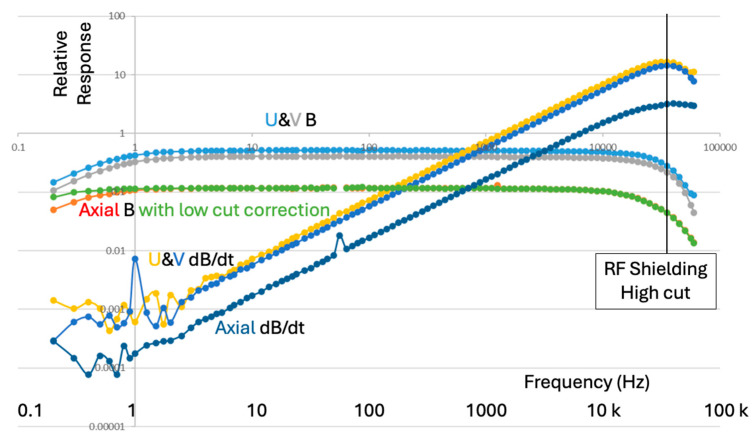
Spectral response of Abitibi Geophysics production prototype axial A and U and V cross-component sensors with both B and dB/dt outputs. The ambient 60 Hz powerline signal is seen at 60 Hz in the Axial component collected by Abitibi Geophysics in Canada. The Abitibi prototype is based on our sensors developed at RMIT University.

The dB/dt response extends well above the resonant frequency of a voltage-measuring coil, with a high-cut corner controlled by the radiofrequency shielding of the carbon-fiber pressure tube. Due to the much smaller absolute amplitudes of the dB/dt signal below 100 Hz, noise dominates at low frequencies in comparison to the B field (Figure 9).

### 4.3. Sensor Comparison

**Figure 10:**
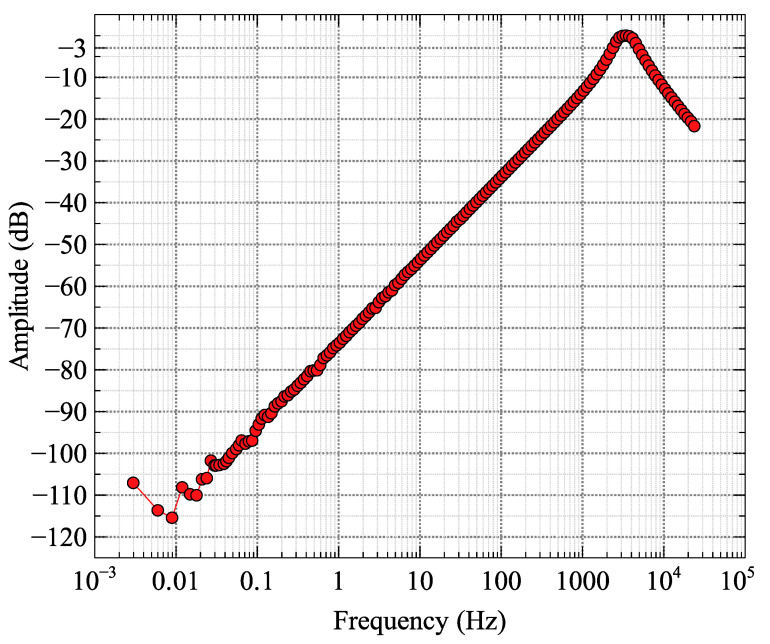
Transfer function for U-V CCIM coil operated as an undamped voltage sensor. The resonant frequency is at 3.42 kHz.

To give us an insight into our improvement to an air-cored coil sensor operated as a voltage sensor, we measured the transfer function of an undamped coil (Figure 10). As evident from the results of Figure 5, Figure 8 and Figure 9, our U-V CCIM reduced the −3 dB corner frequency of a simple current sensor at least 3-fold. The voltage sensor (Figure 10) does not have a low corner, but the large dynamic range leads to unmeasurable small responses from slow EM decays. The peak in response at 3.4 kHz can be changed with damping [21] at the expense of sensitivity, but this plot compared to Figure 9 illustrates how a differentiated current sensor extends the high-frequency bandwidth of a dB/dt measurement by orders of magnitude.

### 4.4. Magnetic Sensor Sensitivity

Using a Helmholtz coil to produce a very uniform field of predictable value, we can determine the sensitivity of magnetic sensors. The magnetic field at the midpoint of a Helmholtz coil is given by

(4) $$B={\left(\frac{4}{5}\right)}^{\frac{3}{2}}\frac{\mu_{0}N_{T}I_{RMS}}{r}\;\left[nT\right],$$

where $\mu_{0}$ is the permeability of free space, r is the distance from the center, $N_{T}$ is the number of turns in each coil, and $I_{RMS}$ is the root mean square input current. Magnetic sensor sensitivity is then measured using the inserting the sensor in the center of the Helmholtz coil then measuring the output sensor voltage and dividing through Equation (3).

Accordingly, the following sensitivity ***S*** values calculated for each of the subscripted U, V, and Axial A sensors are SU=4.53mVnT, SV=3.96mVnT, and SA=11.67mVnT with respective −3 dB low-cut corner frequencies of 5.2 Hz, 7.2 Hz, and 0.1 Hz.

### 4.5. Sensor Noise

Noise estimates for each sensor component were derived from a time series sampled for 30 min inside a shielded Gauss chamber. To determine the sensor’s noise spectral density, we use Welch’s power spectral density estimate as implemented in MATLAB. To convert the voltage amplitude into units of Teslas for the magnetic field, we use the sensitivity values from the previous section. Additionally, we apply a first-order high-pass filter with a corner frequency below the lowest frequency of interest (0.01 Hz) to filter out the effects of any DC offset or low-frequency noise capable of penetrating the shield.

**Figure 11:**
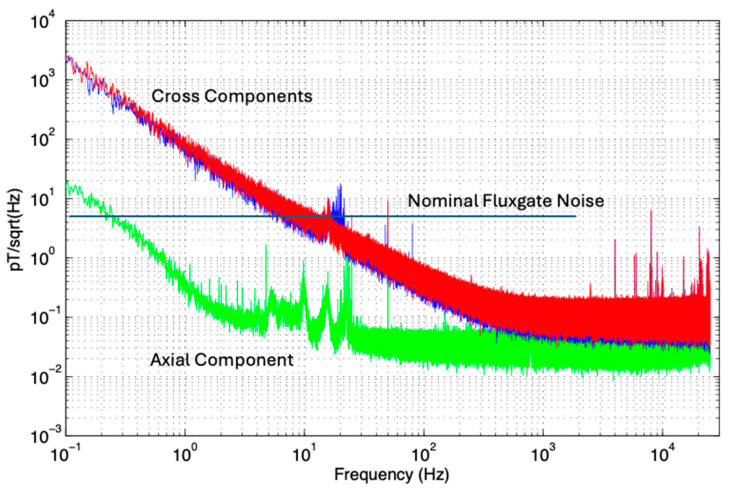
Sequential measurements of shielded chamber noise of the sensors; A (green), U (blue), and V (red) components. External signals from trams (2 to 20 Hz), Australian powerline 50 Hz and harmonics, and high-frequency signals from laboratory lighting and electronics around 10 kHz have not been eliminated by the shielded chamber and appear as spikes above the expected smooth trend of internal sensor noise.

Figure 11 shows an estimate of the internal noise spectrum plus narrow-band leakage signals into the shielded chamber as measured during daytime on a weekday. The anomalous noise in the axial component sensor between 2 and 20 Hz is caused by inner-city trams as evidenced by this noise disappearing between 1 am and 5 am when trams are not running and correlating at non-peak times with the tram schedule. This tram noise has different amplitudes in each of the three components, with the axial component having the greatest S/N ratio. The 50 Hz power line signal and ELF/VLF [42] signal peaks above 3 kHz are evident. The high-frequency peaks were strongly attenuated but not eliminated by the Gauss chamber shielding. Separate runs at different times were used to calibrate the U and V coils, so it is not certain whether the differences in external leakage signal around 20 Hz are due to different sensor orientations or changes in the external signal.

A standard protocol for estimating internal noise is to eliminate uniform external noise by subtracting the simultaneously collected outputs of two sensors aligned in parallel. This subtraction should eliminate any uniform external signals, but the internal uncorrelated noise powers should add rather than subtract. We placed U and V sensors into each of two parallel and adjacent electromagnetic noise-suppressant Gauss chambers and measured background noise using the truncated method presented in [43,44]. Since our sensors have slightly different gains and corner frequencies, a small correction needs to be made in this external signal elimination.

We recorded two time-series signal vectors U and V for our respective sensors inside a solenoid, collected while a wideband transmitter signal was generated. Both sensors should measure the same external signal, and thus, the amplitude scaling factor M between the two signals can be predicted to be

(5) $$M=\frac{V^{T}\cdot U}{V^{T}\cdot V}.$$

If *M* = 1, then the two sensors have identical gain, phase, and orientation in a uniform magnetic field, and a simple subtraction of the two time series would eliminate uniform external signals. In the case M≈1, if the sensors are not quite identical in response or orientation, then the internal noise $C_{UV}$ of each sensor derived from time-series U and V is given by the following equation with the $\sqrt{2}$ factor since the noise powers add when the two internal time series are subtracted:

(6) $$C_{UV}=\frac{MU-V}{\sqrt{2}}$$

**Figure 12:**
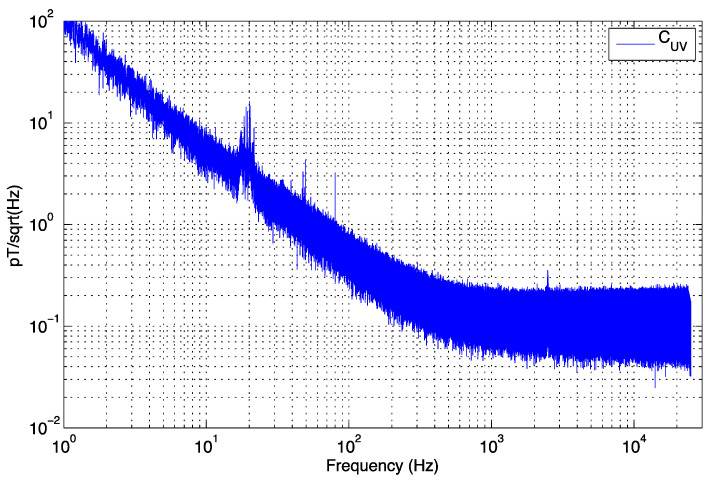
Uncorrelated (internal) noise between sensors U and V placed in parallel and with identical orientation obtained by differencing the sensor outputs after amplitude and phase correction. The remaining peaks above the general trend are attributed to non-stationary noise.

The result of applying spectral analysis to the time series shown in Figure 12 where M=0.94 from Equation (5). Notice the unwanted narrowband signals at high frequencies have been suppressed as opposed to those in Figure 11. The process can be repeated by checking coherence between perpendicular sensors to reinforce everything is working well. Noise suppression across the entire frequency bandwidth can be achieved using the laborious but elegant method of [43,44], summarized in Appendix A, but this approach does not in practice eliminate non-stationary signals such as those in our university laboratory in a central city location. We have therefore attributed locally increased amplitudes in a narrow band of frequencies to unwanted non-stationary signals rather than any inexplicable narrowband internal noise of our sensors.

**Figure 13:**
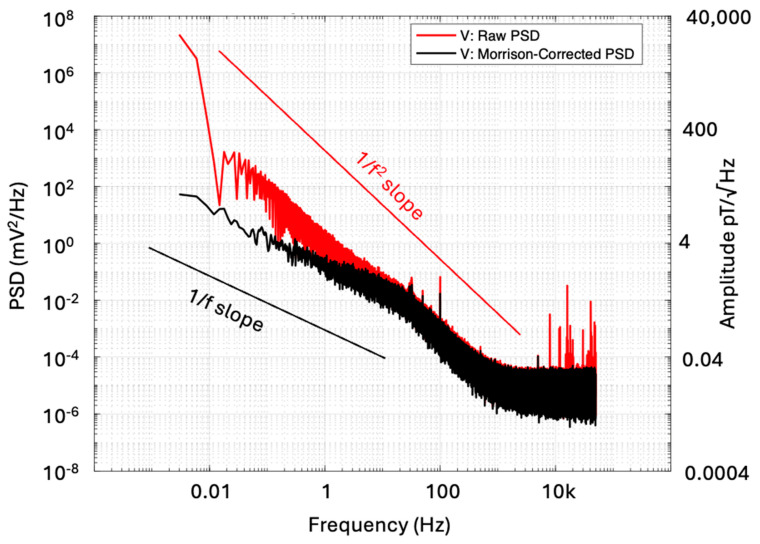
Comparison of raw PSD and Nichols and Morrison-corrected [43,44] PSD for the V component. The corresponding noise amplitude values are shown on the right-hand axis.

Figure 13 compares the raw power spectral density (PSD) with the coherence-corrected PSD for the V component sensor. This correction uses the coherence function γ^2^(f) between U and V sensors to account for potential biases arising from calibration errors, mutual inductance, or crosstalk between the components. It has been suggested in [44] that the coherence between U and V sensors should improve noise estimates, which is clearly performed in this case. Above 100 Hz, we did not find much difference (Figure 13), but there are very significant improvements below 10 Hz.

## 5. Discussion and Conclusions

Our comparison of an optimized coil-induced current-to-voltage sensor with an undamped and un-optimized voltage sensor sensitive to the rate of change in magnetic flux through the coil is not ideal. We did not attempt to optimize a voltage sensor as will have been the case for commercial induction magnetometers using this technology. Our experiments do unambiguously show greatly reduced noise levels compared to the borehole B field fluxgate sensors in current commercial use.

The major engineering challenges of implementing this current sensing technology in pressure-proofed housing with in-hole data collection or communication to the surface are being undertaken by research sponsors Abitibi Geophysics (Canada) and Monex Geoscope (Australia). A field comparison of sensors with available commercial alternatives is the next step once downhole hardware is functional.

A novel borehole cross-component B field sensor using current sensing was assembled and laboratory tested. The novel design achieved a 1.5-decade reduction in corner frequency from a cross-component air-core coil. Signal/noise ratios of the new sensor are much improved compared to alternative borehole technologies. The ancillary dB/dt data available by analog differentiation of the output is linear over a much wider bandwidth than conventional voltage sensors.

## Figures and Tables

**Table 1:** Subjective pros and cons of electromagnetic sensor options for mineral exploration in boreholes at the time research was conducted. Future or recent unpublished sensor technology improvements would affect our pros and cons.

Sensor	Pros	Cons	Reference
Induction coil current sensor (CCIM)	Ideal for axial component; sensitive to long and short decays	Fair for cross-components; reduced long-time constant sensitivity	[21,30]
Feedback coil	Very good for axial component	Limited bandwidth with both low- and high-cut character. Poor for cross-component	[3,21]
Fluxgate	Good for slow decays and very compact so all 3 component sensors are identical	Limited bandwidth insensitive above a few kHz. Higher noise than other sensors above 10 Hz	[31]
Induction coil voltage sensor	Good for fast decays up to a resonant limit. Cross-component a problem	Poor cross-component signal/noise. Poor for slow decays	[7,21]
Analog differentiated current sensor	Best for fast decays. A simple addition to a current sensor	Sensitivity loss at low frequency and poor for slow decays	[30]
MagnetoElectric	Compact sensor with wide bandwidth	Noise levels no better than fluxgates at low frequency	[32]
Tunneling MagneoResistive	Compact	Noise levels orders of magnitude worse than other sensors	[33]
SQUID	Lowest noise	Cryostat will not fit in borehole; venting issue with fluid boiloff	[14]

## Data Availability

Time-series data are available from the authors upon reasonable request.

## Appendix A

Due to the limited online availability of Morrison et al.’s report [43], we provide a summary of his approach to internal and external noise estimation as used with our calibration methodology

Estimates of the noise constituents in electromagnetic data can be obtained by comparing the outputs of two parallel sensors. The noise spectrum of each sensor is computed using its own calibration and auto-power, together with the cross-power of the pair. Assuming that the natural field or laboratory field in adjacent Gauss chambers is uniform over the distance between the sensors, plus assuming the internal noise of a sensor is not coherent with respect to either the natural field or the noise in the other sensor, the cross-spectrum is an internal noise-free determination of the natural field. On the other hand, the auto-power in each sensor is composed of the sum of the external signal and internal noise powers. Stated mathematically, if **A** and **B** are the output spectra from the two (for example our U and V) sensors after calibration correction, **C** is the noise-free natural field spectrum, and **n_A_** and **n_B_** are the internal noise spectra of each of the two sensors, then we can write the following relationships:

E[**AA***] = E[**CC*** + **n**_A_
**n**_A_*] (A1)

E[**BB***] = E[**CC*** + **n_B_ n_B_***] (A2)

E[**BA***] = E[**CC***], (A3)

where E[**AA***] = expected value of [**AA***] and x* = complex conjugate of x.

**A** and **B** can be determined in the frequency domain from observed time-series **U** and **V** from the U and V component sensors, respectively, by dividing their Fourier transforms by **S_0_**, the Fourier transform of the sensitivity time series *S*_0_ given in Equation (1), and measurements in the lab. With digital computers, **U, V**, and **S_0_** should all have the same length and sampling intervals so that their Fourier transforms have identical frequency content. It is also important that the sensitivity response **S_0_** does not have any nulls, limiting this procedure to the useful bandwidth of the sensors used.

The expected values may be estimated by averaging the Fourier spectra from many time records; thus, from Equations (1) and (3), the sensor noise E[**n_A_ n_A_***] for the U component sensor is determined by the difference between the auto-power and cross-power spectra, i.e.,

E[**AA***] − E[**BA***] = E[**n_A_ n_A_***] (A4)

This technique is susceptible to bias caused by errors in the calibration of the sensors. This bias is most severe for measurements when the calibration signal-to-noise ratio is large and may be positive or negative. However, this is mitigated by our reasonable expectation that the calibration involves two single-pole high- and low-cut filters. For example, if we let ε_A_ and ε_B_ be the complex calibration error ε for sensors U and V, respectively, representing errors in magnitude and phase information, then from Equation (A4),

E[**n_A_ n_A_***] = E[**AA***] − E{**BA***]                           = |1 + ε_A_|^2^ [E(**CC***} + E[**n_A_ n_A_***] − (1 + ε_B_) (1 + ε_A_) [E(**CC***]                                = (ε_A_ − ε_B_ − ε_B_ε_A_* + ε_A_ε_A_*) E(**CC***) + (1 − 2Re(ε_A_) + ε_A_ε_A_*) E[**n_A_ n_A_***] (A5)

For cases where coil = amplifier calibrations are known precisely and accurately (as is the case in this paper where calibration measurements can be fitted with single-pole responses), the internal and external noise contributions should be accurately determined. Reference [43] also includes mathematics for the case where the calibration of the sensors is unknown, and can be derived from the data.
